# *Coxiella burnetii* seroprevalence in sheep herd from Paraguay: First evidence of bacterial circulation in the country

**DOI:** 10.1016/j.onehlt.2023.100660

**Published:** 2023-12-10

**Authors:** Danilo Alves de França, Filipe Pereira da Silva, Dayane da Silva Zanini, Lorena Iglesias, Laura Portillo, Herminia Cortez, Alexander Welker Biondo, Ana Íris de Lima Duré, Marcos Vinicius Ferreira Silva, Jorge Miret, Helio Langoni

**Affiliations:** a São Paulo State University, Prof. Doutor Walter Mauricio Correa, s/n, Unesp Campus de Botucatu, Botucatu, São Paulo 18618-681, Brazil; b Octavio Magalhaes Institute, Prof. Octavio Coelho De Magalhaes, s/n, Fundação Ezequiel Dias, Belo Horizonte 30210-290, Minas Gerais, Brazil; c Universidad Nacional de Canindeyú, Calle Itambey, Curuguaty 140802, Paraguay; d Federal University of Paraná, Rua Dr Faivre, 405, Curitiba 140802, Paraná, Brazil

**Keywords:** Q fever, Emerging disease, Small ruminants, Seroepidemiology, Zoonoses, Risk factors, Seroprevalence

## Abstract

*Coxiella burnetii* is the agent of Q fever, a disease that poses risks to public health and damages livestock. We discovered the circulation of *C. burnetii* for the first time in Paraguay, based on the seropositivity of a flock of >300 sheep. The animals were tested by IFA for anti-*C. burnetii* antibodies and by SAM for anti-*Leptospira* spp. antibodies, an important differential diagnosis for reproductive disorders in sheep in Paraguay. *C. burnetii* seropositivity was determined in 45%, in contrast to *Leptospira* spp. which had no reactive samples. Cases of miscarriage and fetal resorption were associated with high seropositivity titers. This study suggests the circulation of a unique genotype in the country and an imminent risk to public health, since in addition to being highly transmissible and infectious to humans, Q fever is still not a cause for concern on the part of government and health agencies in the country.

## 1 Introduction

Coxiellosis or Q fever is a disease caused by a gram-negative bacterium called *Coxiella burnetii*, which causes reproductive symptoms in ruminants and acute flu-like symptoms in humans [1]. Small ruminants in particular are considered the main reservoirs of the disease and sources of infection for humans [2]. Most of the time, the infection is asymptomatic, and the animals eliminate the bacteria without any harm to the pregnancy during parturition, through uterine secretions and the placenta [1]. Although this is the case, the infection can, over time, cause a series of reproductive problems at herd level, such as abortions and infertility, due to an imbalance in the control of the infection and the number of bacteria eliminated into the environment, making not only the herd infected but also herds from neighboring properties [[2], [3], [4], [5], [6]].

Small domestic ruminants are the main sources of infection for humans [7]. Q fever occurs in humans who acquire the infection through infectious aerosols that are eliminated and which, together with dust, travel long distances on the wind [8]. Often, abortion is the only clinical sign of the disease in animals, and since in humans the clinical aspect of the disease involves flu-like symptoms, it goes unnoticed, making it a highly underrated disease [1]. *Coxiella burnetii* has the ability to persist for years in humans, and can lead to endocarditis and other chronic complications [1]. In Latin America, soroprevalence studies have shown the occurrence of the bacteria in populations in Ecuador, Colombia, Argentina, Brazil and French Guiana, mainly in occupational groups [[9], [10], [11]]. In French Guiana, Q fever was reported in 32/131 (24.4%) patients with pneumonia and 25/275 (9.1%) febrile patients [12,13]. In Brazil, 129/604 (21.5%) febrile patients and 4/51 (7.8%) patients with endocarditis were reported [14,15].

A large outbreak of Q fever has already been described in Europe, more precisely in the Netherlands, triggered by sheep, and since then animal soroprevalence and occupational infections have been described in nearby countries [[16], [17], [18]]. In South America, studies carried out in Brazil, Argentina, Chile and French Guiana have pointed to the soroprevalence of the agent in animal herds and human populations, highlighting the possibility that other countries on the continent are being affected by the disease, such as Paraguay, which until now had no reports of human or animal infection [[19], [20], [21], [22]].

Ovine leptospirosis is also reproductive in nature and is one of the main suspected causes of infectious abortion in flocks [[23], [24], [25], [26]]. In South America, given the latest studies published in several countries, leptospirosis should be considered a probable cause of abortion in sheep [27]. In Paraguay, a serological survey which indicated a soroprevalence of 88% in a sheep flock in the district of Yasy Cañy, but without no association with abortions or reproductive failures [28].

An excellent start to uncovering the occurrence of a disease in a country is to investigate it in its main reservoirs. Therefore, the aim of this work was to investigate the presence of antibodies against *Coxiella burnetii* and *Leptospira* spp. in a large sheep flock located in the district of Curuguaty, Paraguay, and to associate this positivity with cases of abortion on the property.

## 2 Methods

### 2.1 Study area and sample collection

A rural property located in the municipality of Curuguaty (24°29′S;55°43′O) in the department of Canindeyú, southeastern Paraguay, was studied. The property had a semi-intensive production method where the animals were fed on feed, hay and pasture. There were around 360 animals and 300 were selected for the study. The samples were selected randomly, all the animals from the first, second and third stables were brought into the enclosure, and all the animals that entered the enclosure were sampled, regardless of breed, age and sex. The animals' reproductive history could not be accessed, however data from the animals' last pregnancy relating to abortions and resorptions was recorded. In the case of breeding animals, cases of orchitis were recorded. Blood samples were collected by certified veterinarians on the property and serum samples were obtained by centrifugation and then stored at −80 °C until laboratory processing.

In order to define a sample size that well represented the population of sheep on the property, a proportional calculation was made using open source epidemiological statistics (Openepi). For the calculation, a population of 360 animals was considered with a precision of 3%, a 95% confidence interval and an anticipated frequency of 50%, since the prevalence of the disease in the region was not known [29].

### 2.2 Serological diagnosis

For *C. burnetii*, serodiagnosis was carried out using an indirect immunofluorescence assay (IFA) developed at the Rickettsioses and Hantaviroses Laboratory of the Octávio Magalhães Institute, Ezequiel Dias Foundation, Brazil. For *Leptospira* spp. serodiagnosis was carried out using the microscopic serum agglutination test (SAM) developed at the Zoonoses Laboratory of the Department of Animal Production and Preventive Veterinary Medicine, São Paulo State University, Brazil.

### 2.3 Coxielosis: indirect immunofluorescence assay (IFA)

The antigen used in the test came from embryonated eggs of the At12 strain, isolated from ticks in Argentina [30]. The sera were tested using anti-ovine IgG conjugate and the positive and negative controls were from sheep previously tested in the laboratory, with positive titres above 1:32.

Aliquots of serum were diluted 1:32 in phosphate-buffered saline (PBS, 0.1 M, pH 7.2) and placed on slides containing the antigens (30ul). The slides were incubated (37 °C for 30 min), washed with PBS and then dried in a humid chamber. Next, 30ul of fluorescein isothiocyanate (FITC)-anti-ovine IgG antibody were added to the concavities, followed by another incubation in a humid chamber for 30 min at 37 °C. After washing and drying again, the slides were mounted with buffered glycerin and coverslips, and read under an immunofluorescence microscope (Olympus BX53) with a 40× objective. Positive samples were subjected to serial dilutions of 1:64, 1:128, 1:256, and so on until the last titer was reached [30].

### 2.4 Leptospirosis: microscopic serum agglutination test (SAM)

A collection of 17 serovars, specific for the diagnosis of herbivores in Latin America, was used. These serovars were stored at 28 °C in Ellinghausen McCullough-Johnson-Harris (EMJH) medium, and included the serovars Bratislava, Bovis, Canicola, Castellonis, Copenhageni, CTG, Djasiman, Grippotyphosa, Guaricura, Hardjo, Icterohaemorraghiae, Mini, Pomona, Prajtino, Pyrogenes, Tarassovi and Wolff.

The sera were diluted with phosphate buffered saline (PBS) (pH 7.6) at a dilution of 1:100, mixed individually with each serovar suspension at a ratio of 1:1 and incubated at 28 °C for 1 h. Pure PBS solution (pH 7.6) was used as a negative control. The analysis was carried out under darkfield microscopy (Carl Zeiss®, Oberkochen, Baden-Württemberg, Germany) at 100× magnification. Samples were considered positive when 50% or more of the leptospires were agglutinated, considering a cut-off titer of 100 [31].

### 2.5 Data analysis

To investigate the risk factors associated with seropositivity in sheep, the data was initially grouped and subjected to a univariate analysis using Pearson's chi-squared test. Using the coefficients obtained for each predictor variable, odds ratios were calculated along with 95% confidence intervals. The most appropriate model was chosen considering the variables that showed significant associations (*p* < 0.05). All tests were carried out using SAS Studio 3.81 (SAS Institute Inc., Cary, NC, USA).

## 3 Results

**Fig. 1:**
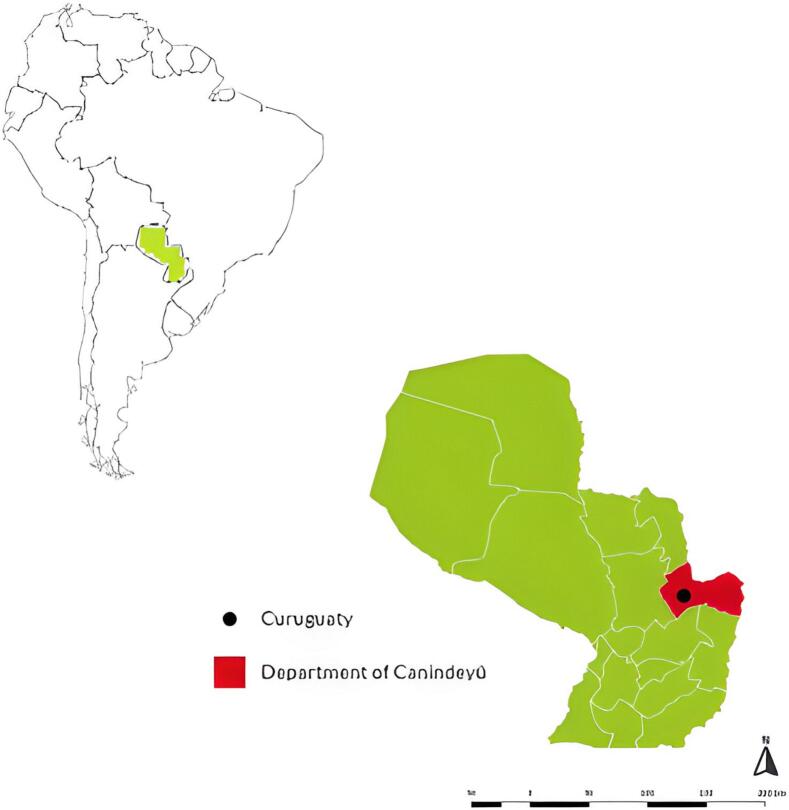
Location of the sheep farm in the municipality of Curuguaty, Paraguay. Map of South America showing the country's location on the continent.

**Fig. 2:**
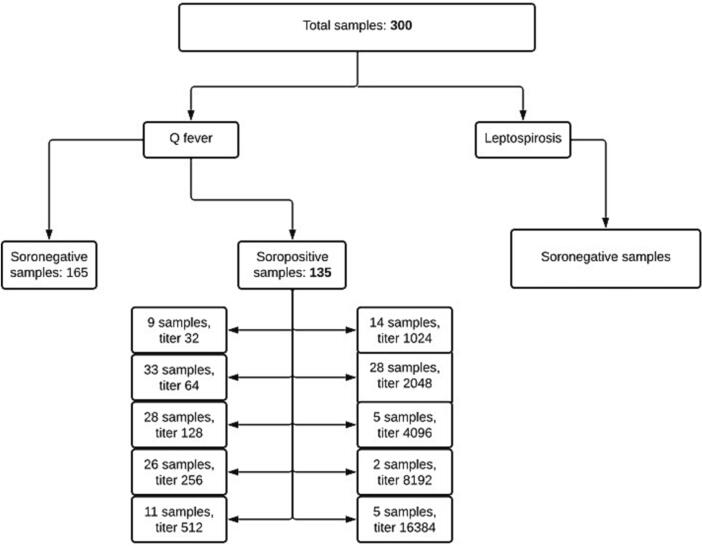
Flowchart of the samples and results of seropositivity.

Seropositive antibodies anti-*C. burnetii* were observed in 135 sheep samples (45%) (95% CI 39.47–50.66), revealing previous exposure to the pathogen in almost half of the flock. There were no reactive samples for *Leptospira* spp. Fig. 2 shows the number of seropositive samples and the titers achieved by these animals. (See Fig. 1.)

The antibody titers achieved by the animals were generally high. The two ewes that aborted on the property were seropositive for *C. burnetii*, one with a titer of 4096 and the other with a maximum detected titer of 16,384. The only female that had fetal resorption was also seropositive, with a titer of 512. As for the males with orchitis, one was seronegative and the other was seropositive with a titer of 64, considered low for a recent active infection. For asymptomatic animals, titres above 800 may indicate persistent chronic infections, which can have repercussions for the producer. In 38.5% of asymptomatic animals, this persistent characteristic can be observed.

Table 1 shows the results of the serological analyses for *C. burnetii* according to the demographic variables collected: breed, age, sex and category. The table shows seropositive and seronegative animals, total population and percentages, allowing an in-depth analysis of those groups described in the literature as more or less likely to acquire the disease.

**Table 1:** Risk factors associated with anti-*Coxiella burnetii* antibodies in sheep herd in the municipality of Curuguaty, Paraguay (2023) by means of univariable statistical analysis.

Variables	*C. burnetii* positive	*C. burnetii* negative	OR (95% CI)	P value	Total population
Breed				0.6356	
Dorper	110 (44.7%)	136 (55.3%)	1.0 (ref)		246
Santa Inês	20 (45.5%)	24 (55.3%)	0.97 (0.51–1.85)		44
Sufolk	6 (60.0%)	4 (40.0%)	0.54 (0.15–1.96)		10
Age				0.0566	
< 1 year old	34 (37.0%)	58 (63.0%)	1.0 (ref)		92
12 to 18 months	26 (63.4%)	15 (36.6%)	0.34 (0.16–0.73)		41
18 to 24 months	14 (40.0%)	21 (60.0%)	0.88 (0.40–1.95)		35
24 to 36 months	15 (53.6%)	13 (46.4%)	0.51 (0.22–1.19)		28
> 3 years old	47 (45.2%)	57 (54.8%)	0.71 (0.40–1.26)		104
Sex				0.2934	
Female	115 (46.7%)	131 (53.3%)	1.0 (ref)		246
Male	21 (38.9%)	33 (61.1%)	1.38 (0.76–2.52)		54
Category				0.4647	
Pregnant	23 (45.1%)	28 (54.9%)	1.0 (ref)		51
With lamb	31 (45.6%)	37 (54.4%)	0.98 (0.47–2.03)		68
Replacement	26 (41.3%)	37 (58.7%)	1.17 (0.55–2.46)		63
Reproducer	21 (38.9%)	33 (61.1%)	1.29 (0.59–2.80)		54
Service	35 (54.7%)	29 (45.3%)	0.68 (0.33–1.43)		64

P value<0.05 indicates statistical difference within the categories. 1.0 (ref.): reference category. OR (95% CI): odds ratio (95% Confidence Interval).

When comparing *C. burnetii* seropositivity with demographic variables, we observed that the Suffolk breed, animals between 12 and 18 months, females and animals in service had higher seropositivity when compared to the other variables in the groups; however, there were no statistically significant differences, as the *P* value for them was >0.05.

## 4 Discussion

The 45% seropositivity (95% CI 39.47–50.66) of anti-*C. burnetii* antibodies in a sheep flock in the municipality of Curuguaty, Paraguay, provides unprecedented data on the circulation of the agent in the country. We couldn't find any previous reports detailing the exposure of sheep to *C. burnetii* in Paraguay. This seroprevalence can be explained by the bacteria's high excreting capacity through birth fluids, the fact that ewes are one of the most susceptible animals and the power of aerosolization, which can easily reach the entire flock when only one animal eliminates the agent [8]. Ticks are considered possible vectors of the bacterium among animals, with more than forty species having been described as being infected [7].

Although there is little data collected on reproductive problems and it is not possible to statistically evaluate an association, the high seropositivity titers in the three cases of reproductive failure in the flock's last pregnancy represent an alarm for the property's reproductive control. Although this is significant, there is also the possibility that there is no relationship between seropositivity and abortion, because as has already been described in Belgium by Djerbib et al. [6], 26.76% of seronegative ewes shed the bacteria through vaginal discharges and feces. According to Anderson et al. [7], a seropositive sheep does not necessarily actively eliminate the bacteria, while a seronegative sheep is able to do so. Direct diagnostics (PCR) of vaginal swabs and abortions need to be carried out to fully understand whether there is a greater chance of these animals having reproductive failures when they become infected. PCR testing of milk or bulk tank milk at farm level also helps to understand whether the animals are excreting the bacteria.

Titers can be used to understand previous exposure and estimate the time when infection occurred [7]. High titers detected in 38.5% of asymptomatic animals indicate a possible persistent infection, which makes it difficult to control the disease on the farm [7]. This study showed no association between demographic variables and seropositivity, differing from a Brazilian study which found a greater chance of seropositivity in male sheep [20]. Semi-intensive sheep farming could also be associated with greater infection in the herd, given that the animals are isolated for long periods of time, which facilitates airborne transmission of pathogens [20].

The seropositivity found in this study was the second highest in farm animals and the highest in sheep in Latin America. Serological surveys in the Latin American countries showed different patterns of seropositivity. Studies with cattle in Brazil and the Caribbean varied in soroprevalence between 0% and 31.8% [32]. With goats, studies in Brazil, the Caribbean, Mexico and Venezuela ranged in soroprevalence from 0% to 60.6% [32]. Studies on sheep showed a much greater variation, with 0% in Argentina, 4.8% in Trinidad, 10.3% in Uruguay and 26.3% in the Caribbean [[33], [34], [35]]. Although high soroprevalence rates have been observed, in most studies these rates ranged between 0% and 3%, as well as having a much smaller sample size [32]. Compared to the highest soroprevalence found, 191/315 (60.6%) of goats from Torres Venezuela [36], this study had a considerably similar sample. In other regions of the world, sheep seroprevalence ranges from 16.5% to 80% [37]. An outbreak of Q fever in Netherlands and an outbreak in Bosnia-Herzegovina showed that sheep, along with goats, are the main source of infection for humans [[38], [39], [40]].

Molecular biology has made it possible to detect the elimination of the agent in the environment, showing a risk to human and herd health, since the bacteria are easily dispersed. The placental membranes and birth fluids are the main routes of elimination of *C. burnetii* by sheep [41]. In southeastern Iran, a study revealed *C. burnetii* positivity by PCR in vaginal fluids in 60/170 (35%) of sheep [42]. In eastern Turkey, *C. burnetii* was detected by PCR in 4/200 (2%) of ovine abortions [43]. In the Iberian Peninsula, a study with a large sample revealed positivity in 483/1241 sheep abortions (38.9%) [44]. More advanced molecular biology studies not only confirm the elimination of the agent, but also point to the emergence of new genotypes in the territories. In Egypt, a molecular typing study characterized *C. burnetii* in four sheep abortions [45]. In Greece, sixty-nine ovine abortion tissue samples were genotyped and showed unique genotypes in Europe [46]. In Brazil, the genotyping of *C. burnetii* in three sheep vaginal swabs revealed a new genotype circulating in Latin America, which, due to its proximity, is believed to be occurring in Paraguay [47].

In addition to vaginal fluids and abortions, milk can also be contaminated in sheep. In southern Italy, *C. burnetii* was detected by PCR in 78/413 (18.9%) raw milk and sheep cheeses [48]. In southeastern Iran, *C. burnetii* was detected by Nested-PCR in 30/170 17.6% milk samples [42]. In northern Spain, *C. burnetii* was detected by real-time PCR in milk from bulk tanks [49]. In Turkey, *C. burnetii* was detected in milk by immunomagnetic separation-PCR [50]. Near Paraguay, in Montería in Colombia, *C. burnetii* was detected by PCR in milk from bulk tanks on 5/11 dairy farms (45%) [51]. In a study carried out in dairy sheep flocks in the Basque Country, Spain, the elimination of *C. burnetii* through milk was observed in several flocks 10 years after its first detection [52]. Bacterial transmission to humans through milk and its derivatives has not yet been proven, but its importance as a potential danger is understood. In Uruguay, Coxielosis was reported on a farm with a history of abortions by *C. burnetii*, which sells and markets artisan cheeses directly to consumers [12].

Leptospirosis is a widely known disease in Paraguay, and along with Brucellosis, it is the main suspected cause of infectious abortions on farms [13]. Other important infectious abortions in sheep are those caused by *Chlamydia abortus*, *Neospora caninum*, and *Campylobacter jejuni* [[53], [54], [55]]. In addition to differential infectious abortion, abortion due to nutritional deficiencies should always be considered [56]. With this study, Coxielosis is presented as an important differential diagnosis for infectious abortions in sheep and a differential diagnosis for leptospirosis in the region's flocks. It is important to develop, on the Paraguayan property of the study, molecular studies that investigate bacterial detection in reproductive tissues, feces and postpartum vaginal discharges and a more in-depth evaluation of the flock's reproductive history so that the disease can be more accurately associated with loss of animal production.

Herd infection can remain active for many years if effective control and biosecurity measures are not implemented correctly, such as correct managing maternity wards, e.g. The management of maternity wards should serve to prevent environmental contamination from posing a risk to infection-free animals at the most critical time for controlling the disease, which is postpartum. Standard abortion control measures, including the immediate removal of aborted material, the segregation of animals by pregnancy status and the diagnostic evaluation of abortions, are all guaranteed. Environmental control measures are also recommended, keeping parturient animals downwind from the rest of the herd or housed in a controlled ventilation area and minimizing dusty conditions in the facilities. The use of vaccines based on phase 1 antigens should be considered in this context and their eligibility studied within the context of South America.

## 5 Conclusions

To our knowledge, this is the first report of *Coxiella burnetii* infection in Paraguay. This study revealed the presence of *C. burnetii* in domestic sheep in southeastern Paraguay. The sheep showed no seroreactivity to *Leptospira* spp. Two cases of abortion on the property and one case of fetal resorption were associated with high antibody titers to *C. burnetii*. The high soroprevalence of Coxielosis indicates that the animals have been previously exposed to the bacteria. More studies are needed to better characterize the epidemiology of Q fever infection in animals and humans in Paraguay.

## Funding

This work was supported by the 10.13039/501100001807São Paulo Research Foundation (FAPESP) grant number 2022/07124-6.

## Declaration of Competing Interest

The authors have declared that no competing interests exist.

## Data Availability

All relevant data are within the manuscript.
